# Author Correction: Pathway to a land-neutral expansion of Brazilian renewable fuel production

**DOI:** 10.1038/s41467-022-31235-1

**Published:** 2022-06-15

**Authors:** Luis Ramirez Camargo, Gabriel Castro, Katharina Gruber, Jessica Jewell, Michael Klingler, Olga Turkovska, Elisabeth Wetterlund, Johannes Schmidt

**Affiliations:** 1 Institute for Sustainable Economic Development, University of Natural Resources and Life Sciences, Vienna, Austria; 2 Electric Vehicle and Energy Research Group (EVERGI), Mobility, Logistics and Automotive Technology Research Centre (MOBI), Department of Electrical Engineering and Energy Technology, Vrije Universiteit Brussel, Brussels, Belgium; 3 Copernicus Institute of Sustainable Development, Utrecht University, Utrecht, The Netherlands; 4 Energy Planning Program, Graduate School of Engineering, Universidade Federal do Rio de Janeiro, Rio de Janeiro, Brazil; 5 Department of Space, Earth and Environment, Chalmers University of Technology, Gothenburg, Sweden; 6 Center for Climate and Energy Transformations and Department of Geography, University of Bergen, Bergen, Norway; 7 International Institute for Applied Systems Analysis (IIASA), Laxenburg, Austria; 8 Department of Geography, University of Innsbruck, Innsbruck, Austria; 9 Energy Engineering, Division of Energy Science, Luleå University of Technology, Luleå, Sweden

**Keywords:** Energy and society, Environmental social sciences

Correction to: *Nature Communications* 10.1038/s41467-022-30850-2, published online 07 June 2022.

The original version of this Article contained an incorrect reference to the database used in the study (reference no. 73). This has been corrected in both the PDF and HTML versions of the Article.

