# Supplementary material for: Pathway to a land-neutral expansion of Brazilian renewable fuel production
Source: Nat Commun. 2022 Jun 7;13:3157. doi: 10.1038/s41467-022-30850-2 (PMC9174478; doi:10.1038/s41467-022-30850-2)
Supplement: Supplementary file 1 — Supplementary Information [file 41467_2022_30850_MOESM1_ESM.pdf]

Supplementary Information - Pathway to a land-neutral expansion of Brazilian renewable fuel production

Luis Ramirez Camargo[1,2,3*], Gabriel Castro[1,4], Katharina Gruber[1], Jessica Jewell[5,6,7], Michael Klingler[1], Olga Turkovska[1], Elisabeth Wetterlund[8,7], Johannes Schmidt[1*]

[1]*Institute for Sustainable Economic Development, University of Natural Resources and Life Sciences, Vienna, Austria*
[2]*Electric Vehicle and Energy Research Group (EVERGI), Mobility, Logistics and Automotive Technology Research Centre (MOBI), Department of Electrical Engineering and Energy Technology, Vrije Universiteit Brussel, Brussels, Belgium*
[3]*Copernicus Institute of Sustainable Development, Utrecht University, Utrecht, The Netherlands*
[4]*Energy Planning Program, Graduate School of Engineering, Universidade Federal do Rio de Janeiro, Rio de Janeiro, Brazil.*
[5]*Department of Space, Earth and Environment, Chalmers University of Technology, Gothenburg, Sweden*
[6]*Center for Climate and Energy Transformations and Department of Geography, University of Bergen, Bergen, Norway*
[7]*International Institute for Applied Systems Analysis (IIASA), Laxenburg, Austria*
[8]*Energy Engineering, Division of Energy Science, Luleå University of Technology, Luleå, Sweden*

Supplementary Figures

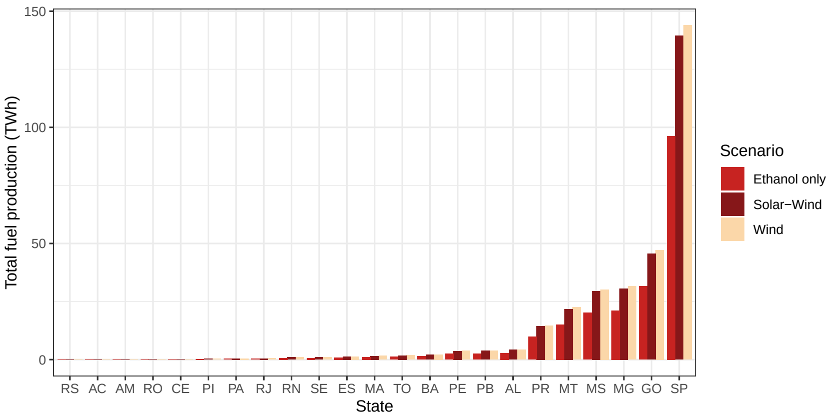

Supplementary Fig. 1: Total production of renewable fuels per Brazilian state. RS, Rio Grande do Sul; AC, Acre; AM, Amazonas; RO, Roraima; CE, Ceará; PI, Piauí; PA, Paraná; RJ, Rio de Janeiro; RN, Rio Grande do Norte, SE, Sergipe; ES, Espirito Santo; MA, Maranhão; TO, Tocantins; BA, Bahia; PE, Pernambuco; PB, Paraiba; AL, Alagoinhas; PR, Paraná; MT, Mato Grosso; MS, Mato Grosso do Sul; MG, Minas Gerais; GO, Goiania; SP, São Paulo.

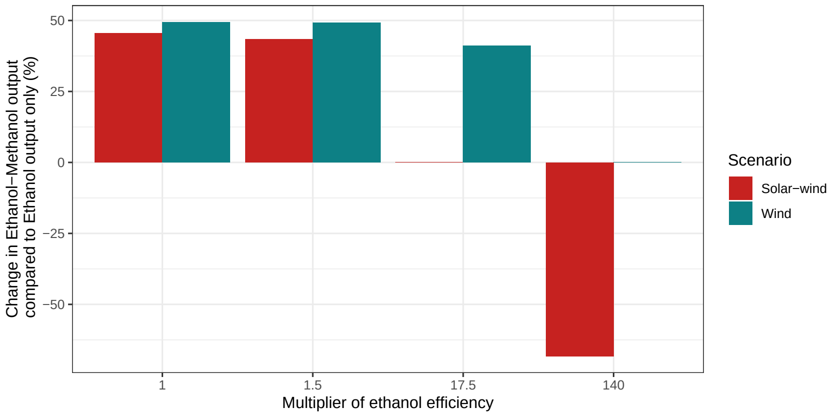

Supplementary Fig. 2: Change in output of the land-neutral methanol pathway in different ethanol land-use efficiency scenarios. The x-axis shows a multiplicator of current ethanol efficiency. The y-axis shows by how much the methanol pathway can increase total fuel output in energetic terms. E.g. if current ethanol efficiency is multiplied by 17.5, the methanol pathway does not offer any additional production potential in the solar-wind scenario

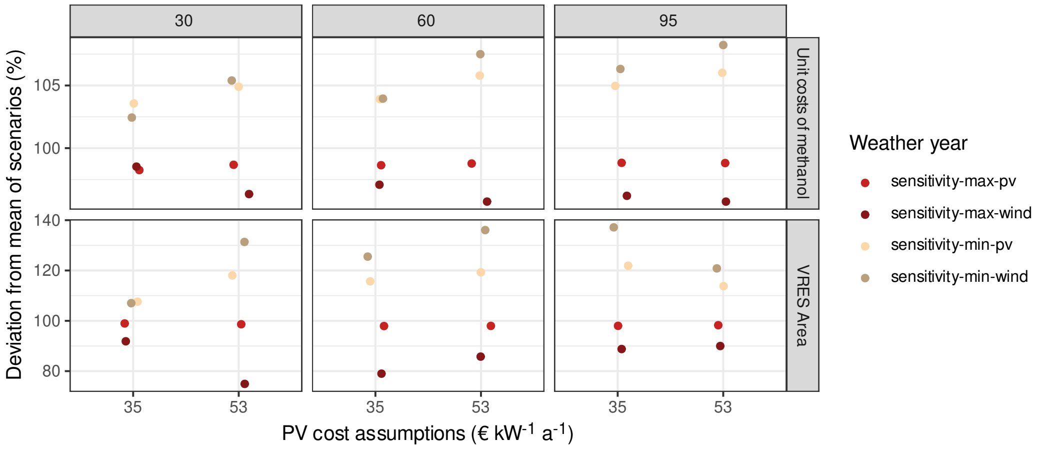

Supplementary Fig. 3: The impact of different weather years on VRES area and methanol cost for different electrolyzer cost assumptions From left to right: 30, 60 and 95 € kW^{-1} a^{-1} annualized electrolyzer cost. PV, Photovoltaics

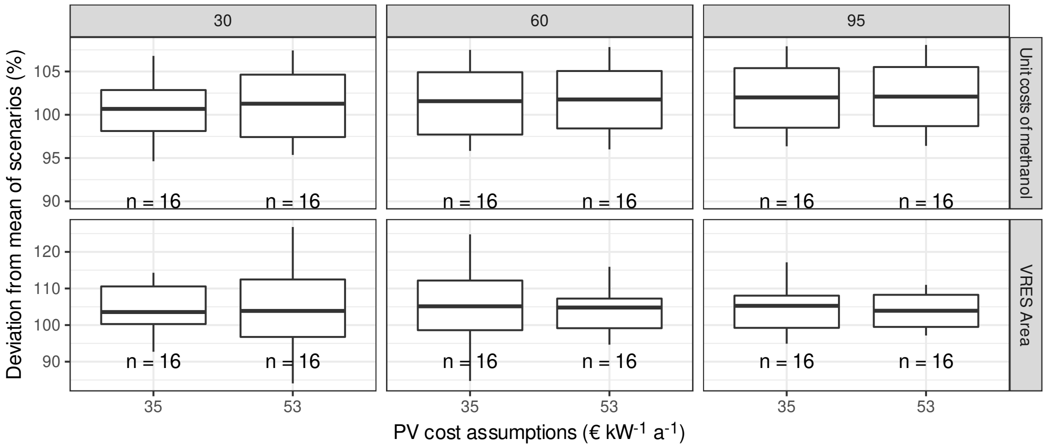

Supplementary Fig. 4: The impact of storage, and wind power cost and electrolyzer efficiency assumptions on methanol cost and land-use for different electrolyzer and pv cost scenarios. Each boxplot shows 16 different combinations of battery storage, H_2 storage, and wind power cost, and electrolyzer efficiency assumptions. From left to right: 30, 60 and 95 € kW^{-1} a^{-1} annualized electrolyzer cost. The CO_2 storage cost is fixed at 138€ $tCO_2^{-1}a^{-1}$ in all shown scenarios. Horizontal lines in the boxes represent the medians, the upper and lower boundary of the boxes represent the 25th (bottom hinge) and 75th (top hinge) percentiles. The top/bottom whiskers reflect the maximum/minimum if there are no outliers; in cases of the existence of outliers (i.e. if there are values below the 25th percentile - 1.5 times the interquartile range or values above the 75th percentile + 1.5 the interquartile range), the whiskers represent the highest/lowest values within 1.5 times the interquartile range. The outliers are then shown as single points. PV, Photovoltaics; VRES, Variable Renewable Energies

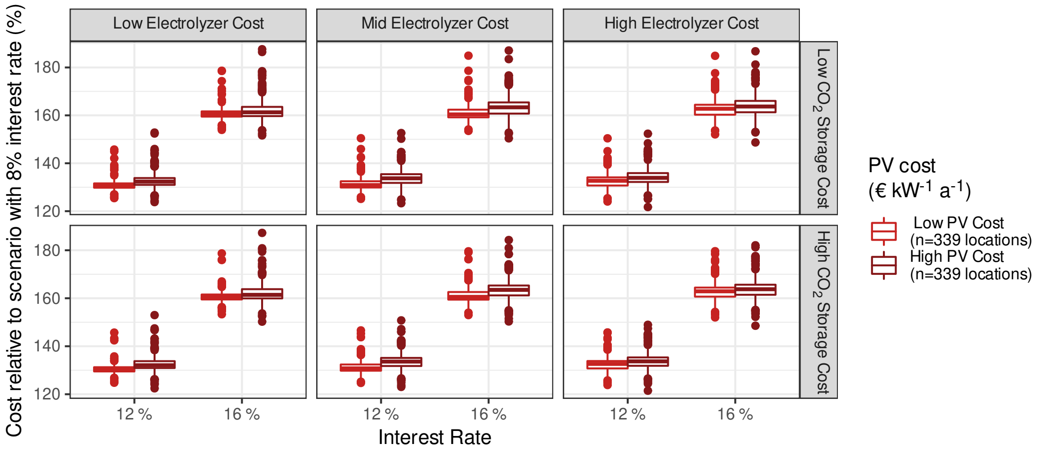

Supplementary Fig. 5: The impact of the chosen interest rate on methanol cost for all locations. The boxplots show individual locations for a given scenario. The electrolyzer efficiency is chosen to be 69% and we show the mixed PV and wind scenario here. Horizontal lines in the boxes represent the medians, the upper and lower boundary of the boxes represent the 25th (bottom hinge) and 75th (top hinge) percentiles. The top/bottom whiskers reflect the maximum/minimum if there are no outliers; in cases of the existence of outliers (i.e. if there are values below the 25th percentile - 1.5 times the interquartile range or values above the 75th percentile + 1.5 the interquartile range), the whiskers represent the highest/lowest values within 1.5 times the interquartile range. The outliers are then shown as single points. PV, Photovoltaics

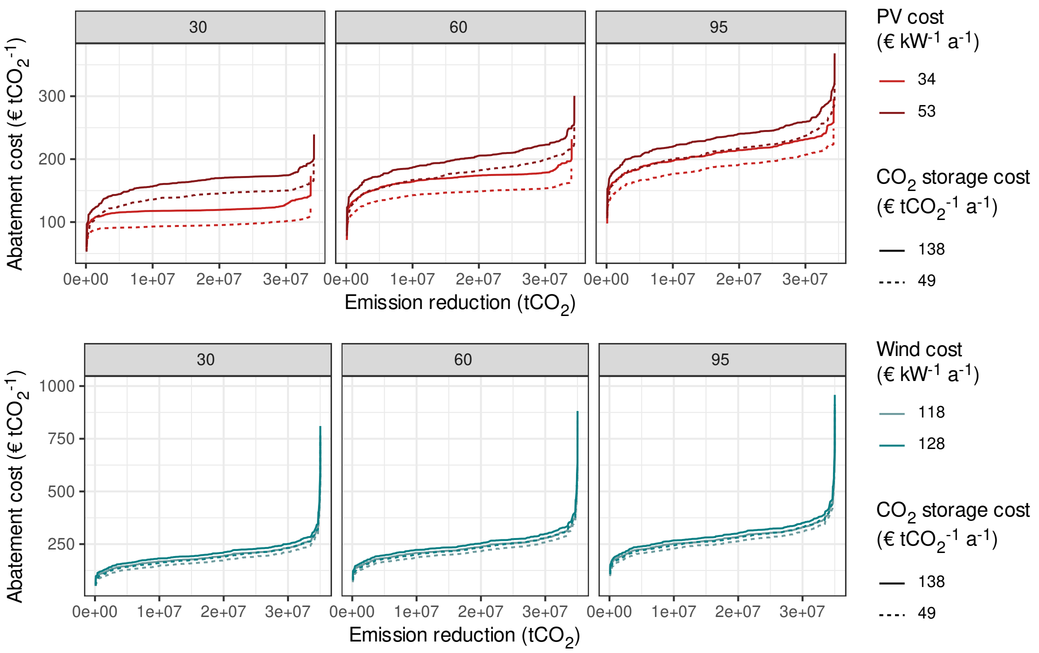

Supplementary Fig. 6: CO_2 abatement cost curves. Upper: solar-wind scenario. Lower: wind scenario. From left to right: annualized electrolyzer cost assumptions (€ kW^{-1} a^{-1}). PV, Photovoltaics

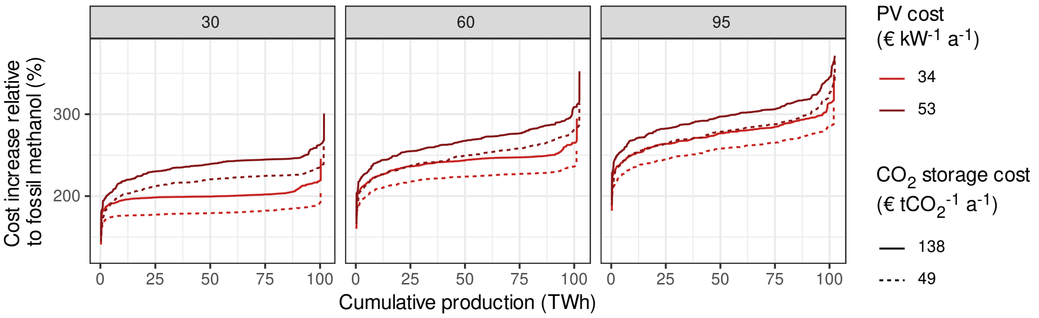

Supplementary Fig. 7: Relative cost of methanol in land-neutral pathway compared to fossil methanol cost. The solar-wind scenarios are shown. From left to right: 30, 60 and 95 € kW^{-1} a^{-1} annualized electrolyzer cost. PV, Photovoltaics

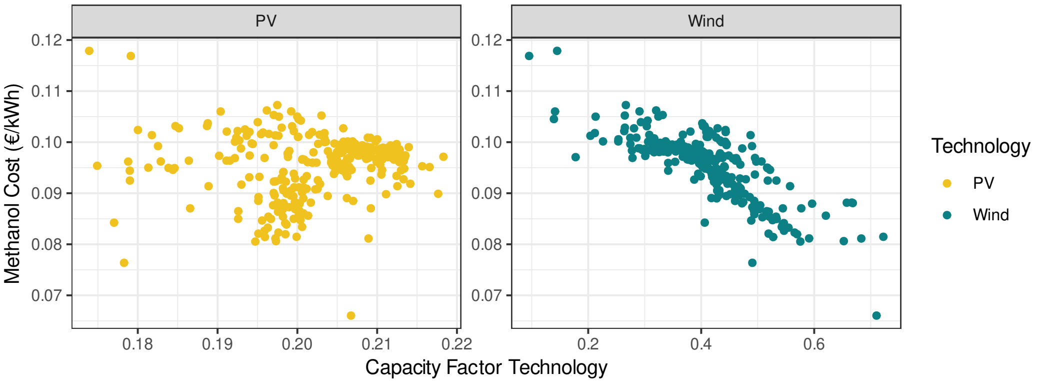

Supplementary Fig. 8: Full load hours of wind and PV vs. methanol production cost in a selected solar-wind scenario. PV, Photovoltaics

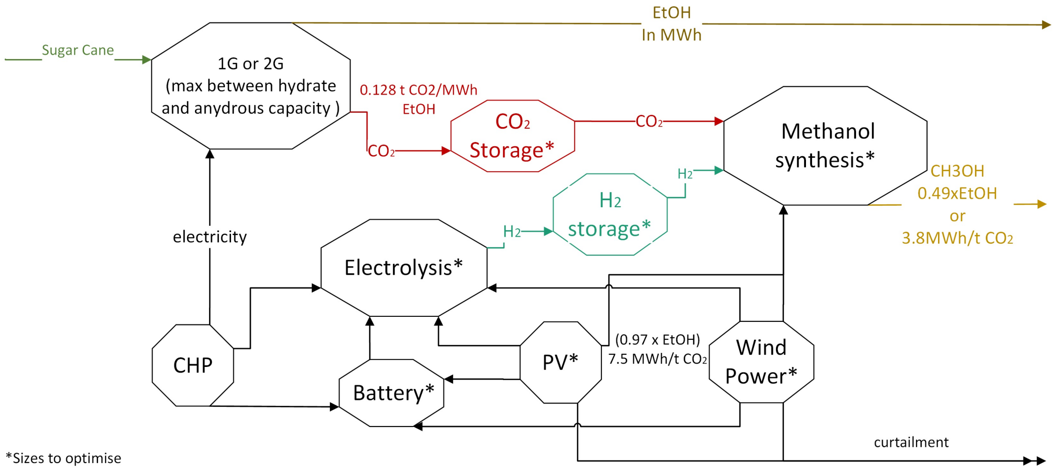

Figure 9: Schematic presentation of the processes modelled in all sugarcane to ethanol and methanol installations. 1G, First generation ethanol production technology; 2G, Second generation ethanol production technology; CHP, Combined heat and power; PV, Photovoltaics

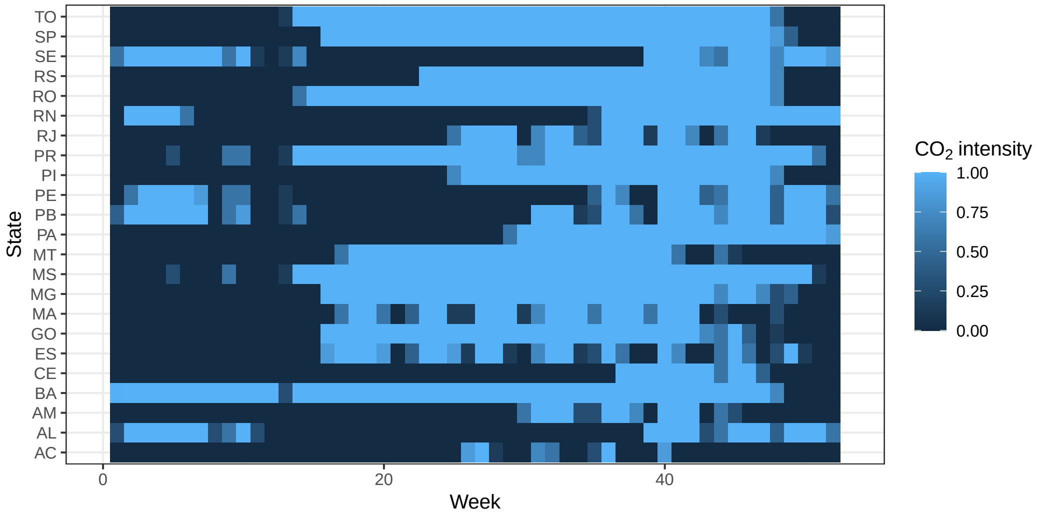

Supplementary Fig. 10: Heatmap of CO_2 streams from sugarcane fermentation for all states, normalized by maximum production per state. RS, Rio Grande do Sul; AC, Acre; AM, Amazonas; RO, Roraima; CE, Ceará; PI, Piauí; PA, Paraná; RJ, Rio de Janeiro; RN, Rio Grande do Norte, SE, Sergipe; ES, Espirito Santo; MA, Maranhão; TO, Tocantins; BA, Bahia; PE, Pernambuco; PB, Paraiba; AL, Alagoinhas; PR, Paraná; MT, Mato Grosso; MS, Mato Grosso do Sul; MG, Minas Gerais; GO, Goiania; SP, São Paulo

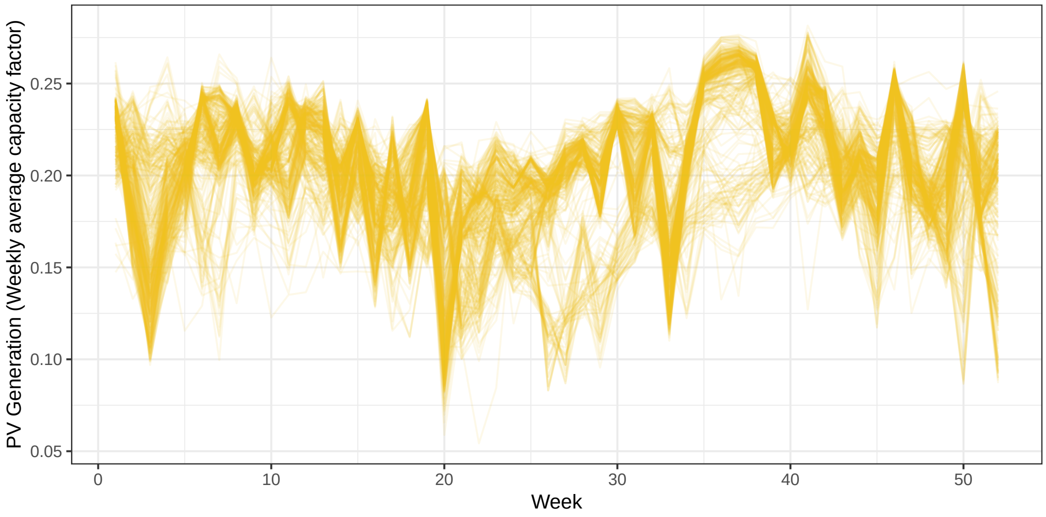

Supplementary Fig. 11: Average weekly capacity factor of solar PV installations at all locations. PV, Photovoltaics

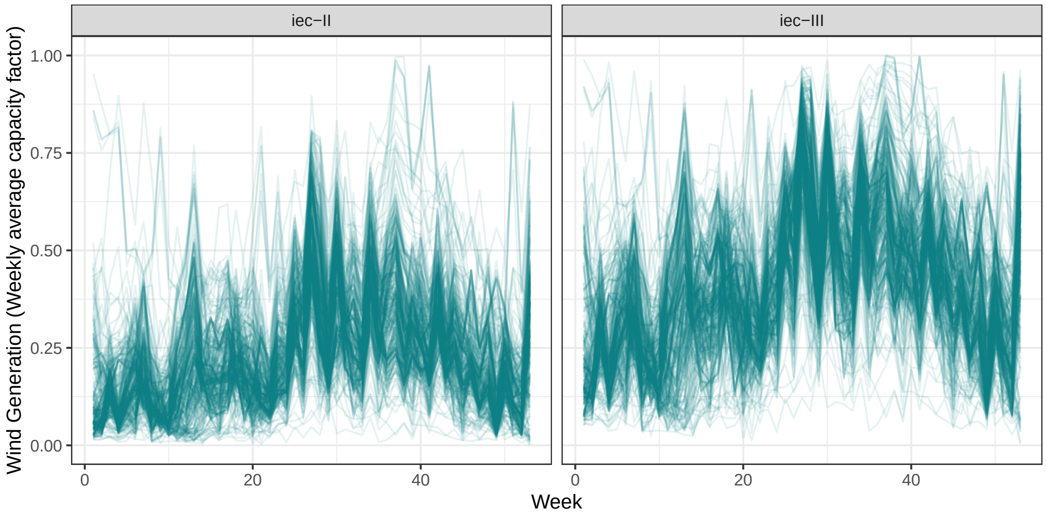

Supplementary Fig. 12: Wind power generation time series at all locations. Left: time series for IEC-II turbine, right: time series for IEC-III turbine. IEC, International Electrotechnical Commission.

Supplementary Tables

Supplementary Table 1: Technology assumptions

Technology	CAPEX (€ kW^{-1})	OPEX (€ kW^{-1} a^{-1})	Lifetime (years)	Annualized cost (€ kW^{-1} a^{-1})	Efficiency	Source
PV						Cost projection for
Low	290	8.8	20	34	-	2020 (high) and 2030 (low)
High	431	6,4	20	53		are taken from[1]
Wind						Cost projection for 2020(high) and 2030(low)
Low	1,040	12	20	118	-	are taken from[2].
High	1,120	14	20	128		OPEX are taken from[3]
Electrolyzers						The selected values are a conservative summary
Low	250	5.0	20	30	63%	of the references provided
Mid	500	10.0	20	60		in Supplementary Table 2.
High	780	15.7	20	95	69%	OPEX and efficiency from[3]
Methanol synthesis	300	12	20	43	83% (from H_2)	[4]
	**CAPEX (€ t^{-1})**	**OPEX (€ t^{-1} a^{-1})**	**Lifetime (years)**	**Annualized cost (€ t^{-1} a^{-1})**		
H_2-Storage*						
Low	82,423	1,033	20	7,979	100%	[5]
High	1,171,863	1,155	20	74,302		
CO_2-Storage						
Low	381	10	20	49	100%	[6, 7, 8]
High	1402	451	20	138		[9, 10, 11]
	**CAPEX (€ kWh^{-1})**	**OPEX (€ kWh^{-1} a^{-1})**	**Lifetime (years)**	**Annualized cost (€ kWh^{-1} a^{-1})**		
Battery						Low and high OPEX and
Low	117	2.7	15	16	90%	CAPEX cost represent estimates taken from[1] for 2020
High	250	3.9	15	33		and 2030, respectively.

*Annualized costs were not directly calculated from CAPEX and OPEX, for details please see the Excel file "techno-economic-parameters.xlsx" in the zenodo repository (`https://doi.org/10.5281/zenodo.6471331`)

Supplementary Table 2: Electrolyzer cost reported in literature (converted from USD to € for an average exchange rate of 1.142 USD €$^{-1}$)

Electrolyzer type [year]	Price (range) [€ kW^{-1}]	Reference
Alkaline [2020]	500-1,110	[12]
PEM [2020]	337-1,811	[12]
PEM [2020]	701	[13]
PEM [2030]	263	[13]
Generic [2020]	438-832	[14]
Generic [2030]	350-832	[14]
Generic [2040/current Chinese low cost]	175	[15]

Supplementary Table 3: CO_2 capture cost from ethanol production reported in literature

Reference Year	Price (range) [€ tCO_2^{-1}]	Reference
2020	<10	[16]
2017	Close to 0	[17]
2017	<8	[18]

Supplementary Table 4: Techno-economic assumptions in scenarios shown in Fig.2 and used in the regression analysis

**Parameter**	**Value**	**Unit**
Electrolzyer Cost	60	€ kW^{-1} a^{-1}
Electrolyzer Efficiency	0.69	-
PV Cost	34	€ kWp^{-1} a^{-1}
Wind Power Cost	128	€ kW^{-1} a^{-1}
CO_2-Storage Cost	138	€ t^{-1} a^{-1}
H_2 Storage Cost	33	€ kWh^{-1} a^{-1}
Battery Storage Cost	74,000	€ t^{-1} a^{-1}
Weather year	2016	-

Supplementary Table 5: Results of regression model fitting model input parameters on cost of methanol production (€ kWh^{-1}) using Ordinary Least Squares. We used the solar-wind scenario with the highest input cost assumptions. The p-values are derived from a two sided t-test. The exact p-values are 2.17e-119, 4.76e-01, 4.39e-102 and 6.35e-51 for the Intercept, $\overline{\text{PV}}$, $\overline{W}$, and l^{season} respectively

	**Methanol cost (€ kWh^{-1})**			
Predictors	***Estimates***	***Std-Error***	***t-value***	***p-value***
Intercept	0.1364	0.004	36.6922	<0.001
$\overline{\text{PV}}$	-0.0126	0.0176	-0.7138	0.4758
$\overline{W}$	-0.0508	0.0016	-31.4738	<0.001
l^{season}	-0.0001	0.0000	-17.9425	<0.001
**Observations**	339			
**R^2 / R^2 adjusted**	0.82 / 0.82			

Supplementary Table 6: Optimization model sets

**Name**	**Symbol**	**Unit**	**Elements**
Time steps	t	h	t_1,t_2,...,t_n
commodity	c	-	electricity, CO_2, H_2
technology	*tech*	-	pv, wind
Sub-technology	*subtech*	-	IEC-II, IEC-III

Supplementary Table 7: Optimization model parameters

	**Name**	**Symbol**	**Unit**	**Value**
Time Series	Electric energy generation of VRES	vresOutput(t, tech, subtech)	GWh	Variate per time step and location (see methods section)
	CO_2 generated in the ethanol production	co2Streamt	t	
Cost	Cost of VRES technologies	vresCost(tech, subtech)	€ GW^{-1}	See Supplementary Table 1.
	Cost of the electrolyzer	electrolyzerCost	€ GW^{-1}	See Supplementary Table 1.
	Cost of the methanol synthesis system	methanolSynthesisCost	€ GWh^{-1}	See Supplementary Table 1.
	Cost of storage	storageCostc	€ GWh^{-1} and € t^{-1}	See Supplementary Table 1.
Efficiencies and commodities transformation balances	Efficiency of storage	storageEffcharg$_c$	%	See Supplementary Table 1.
	Losses of the storage system from one period to the next	storageLosst	%	electricity=0.1<br>CO_2=0<br>H_2=0
	Charge and discharge capacity of storage systems	storageCapacityLimitPercentagec	%	electricity=40<br>CO_2=20<br>H_2=20
	Balance between CO_2 and H_2 for each Methanol unit	balanceCO2H2	-	7.268519[4]
	Electrolyzer efficiency	h2Eff	kt GWh^{-1} (LHV)	See Supplementary Table 1.
	Methanol synthesis efficiency	methanolSynthesisEff	GWh kt^{-1} (LHV)	28.21[4]

Supplementary Table 8: Optimization model variables

Name	Symbol	Unit
Total annualized system costs	x_cost	€
Total VRES generation	$x\_vresGeneration_t$	GWh
VRES installed capacity	$x\_vresBuild_{(tech,subtech)}$	GW
VRES electricity with immediate use	$x\_vresPowerToUse_t$	GWh
VRES electricity to store	$x\_vresToStorage_t$	GWh
VRES electricity to curtail	$x\_vresCurtail_t$	GWh
Storage system input	$x\_storageInput_{(t,c)}$	GWh or kt
State of charge of storage	$x\_soc_{(t,c)}$	GWh or kt
Storage system output	$x\_storageOutput_{(t,c)}$	GWh or kt
Storage system size	$x\_storageSize_c$	GWh or kt
Electrolyzer size	x_electrolyzerSize	GW
H_2 produced	x_h2_t	GWh
Methanol Synthesis	x_methanolSynthesisSize	GW
Methanol produced	$x\_methanol_t$	GWh
Electricity to produce H_2	$x\_electricityGenerationH2_t$	GWh
CO_2 to methanol	x_co2ToMethanol	kt
H_2 to methanol	x_h2ToMethanol	kt

Supplementary Table 9: Footprints of photovoltaic installations at seven locations in Brazil

Plant	State	Capacity (MW)	Year of installation	lon	lat	Area (m^2) with spacing	Area (m^2) without spacing	m^2 kWp^{-1} with spacing	m^2 kWp^{-1} without spacing
Nova Aurora	SC	3.07	2013	-48.97	-28.45	44,490	40,625	14.5	13.2
Tanquinho	SP	1.08	2012	-47.04	-22.88	18,964	13,642	17.5	12.6
Apodi I–IV	CE	132	2018	-37.79	-5.04	4,050,000	1,178,998	30.7	8.9
Floresta I–III	RN	86	2017	-36.91	-4.96	2,900,000	750,037.5	33.7	8.7
Guimarania 1 + 2	MG	62	2018	-46.67	-18.82	1,807,653	819,383.5	29.2	13.2
Assú V	RN	30	2017	-37.03	-5.55	873,546	306,397	29.1	10.2
Guaimbé 1–5	SP	150	2018	-49.87	-21.89	2,250,000	1,801,319	15.0	12.0

Supplementary Table 10: Descriptive statistics of footprints of PV installations in Brazil

Quantile	m^2 kWp^{-1} with spacing	m^2 kWp^{-1} without spacing
0	14.50	8.72
25%	20.42	9.25
50%	29.16	10.21
75%	29.16	12.01
100%	33.72	13.22
mean	24.2	11.3

Supplementary Table 11: Footprints of photovoltaic installations reported in the literature

Given unit	Land requirement [m^2 kWp^{-1}]	System type	Source
	**with spacing**		
35 W m^{-2}	28.6	land use	[19]
65 W m^{-2}	15.4	25°tilt south panel, USA	[20]
48 W m^{-2}	20.8	1-axis tracking panel, USA	[20]
20 W m^{-2}	50	2-axis tracking panel, USA	[20]
7.5 acres $MWac^{-1}$	30.4	total LU large PV, fixed	[21]
8.3 acres $MWac^{-1}$	33.6	total LU large PV, 1-axis	[21]
8.1 acres $MWac^{-1}$	32.8	total LU large PV, 2-axis CPV	[21]
5.8 acres $MWac^{-1}$	23.5	direct LU large PV, fixed	[21]
9.0 acres $MWac^{-1}$	36.4	direct LU large PV, 1-axis	[21]
6.1 acres $MWac^{-1}$	24.7	direct LU large PV, 2-axis CPV	[21]
	**Without spacing**		
1.4 ha MWp^{-1}	14	area	[22]
7.1 m^2 kWp^{-1}	7.1	panel	[23]
135 W m^{-2}	7.3	flat panel (rooftop), USA	[20]
118 W m^{-2}	8.5	10°tilt south panel (rooftop), USA	[20]

Supplementary note 1. Results - Sensitivity analysis

Here, we assess the sensitivity of model results to weather years (Supplementary Fig. 3) and storage and wind power cost (Supplementary Fig. 4). We ran the model for three different weather year configurations: first, for our reference year 2016. Average PV electricity generation in this year is closest to the average annual PV electricity generation in the period 1999-2018. Second we simulated the years with the lowest and with the highest annual wind and PV generation. For these runs, the lowest/highest generation years were defined by the individual minimum or maximum production years in the whole time series of renewable generation of 20 years (1999-2018) per location. Therefore, the optimization model was run for three different years at all locations: for 2016, for the year with the lowest and for the year with the highest generation at that location. This is, of course, a rather extreme assumption, as the best years are not uniform among regions. In this way, however, we were able to cover the most extreme impacts of climate on results.

Although we took a rather extreme approach, the impacts on both of our key performance indicators, i.e., land used by renewables and final cost, were minor. The average cost in all scenarios is in the range of 96% to 108% of the baseline scenario. For land-use, this range is wider from 75% to 137%, as land-use is strongly affected by changes of the wind power share in total generation. This indicates that at some locations, portfolios with different shares of solar PV and wind power are comparable in total cost.

The impact of different H_2 storage and wind power cost assumptions and the electrolyzer efficiency (see Supplementary Table 1) on overal cost is limited (Supplementary Fig. 4). Methanol cost varies in the range of 95% to 108%. We assessed which factors contributed most and found that the highest impact has a drop in the electrolyzer efficiency as we assess differences in 10% (6 percentage points) of conversion efficiency. The other parameters are of minor relevance: very low levels of H_2 storage are deployed, the cost assumption is therefore less relevant for total cost. Our variation of wind power cost assumptions is low, as we consider it to be a mature technology. The minor changes in cost do consequently not affect output strongly. For land-use, the sensitivity analysis shows a wider range of possible outcomes of 84% to 127% of mean land-use, depending on parameter choice. This confirms that PV and wind power are closely competitive at many locations, and a minor change in cost parameters can cause a significant shift in the wind power share and therefore in land-requirements.

We also assessed the sensitivity of our results to changing interest rates (Supplementary Fig. 5). When increasing the interest rate from 8% to 12% and 16%, cost on average increases by 33% and 62%. However, there is quite some variation within scenarios, caused by changes in the wind power share at some locations. The impact of the interest rate on the relative cost of wind power production to solar PV will depend on local wind resources. Higher interest rates exacerbate competitive difference between technologies, i.e. a change in the interest rate can both increase and decrease the wind power share. Therefore, the relative change in cost at different locations can show substantial variation. A decrease in the wind share will increase the relative cost increase, and vice versa.

Supplementary note 2. Results - Abatement cost and comparison to fossil methanol

We calculated CO_2 abatement cost by assuming that renewable methanol substitutes methanol produced from fossil natural gas at an emission factor of $335.8\,tCO_2\ GWh^{-1}$ [24]. We assumed a cost of methanol produced from fossil gas of 0.04€ kWh^{-1} [25], sorted all locations by production cost from lowest to highest, and calculated the potential abated emissions by the sum of the production times the emission factor. Abatement cost was calculated as the difference of renewable production cost and fossil production cost divided by the emission factor. These are presented in Supplementary Fig. 6. Furthermore, we show the relative cost increase of methanol produced in our pathway to fossil methanol, assuming a cost of 0.04€ kWh^{-1} (Supplementary Fig. 7).

Supplementary note 3. Results – Explaining cost of methanol

Supplementary Fig. 8 shows how the full load hours of wind and PV impact the methanol production cost for the solar-wind scenario. Higher PV full load hours do not consistently lower production cost, also as the variation is relatively low, while wind power does – in particular for capacity factors above 0.4. We also developed a simple regression model, which tests the influence of input parameters on methanol production cost:

$$c^{\text{methanol}} = \beta_0 + \beta_1 \overline{\text{PV}} + \beta_2 \overline{W} + \beta_3 l^{\text{season}}$$

We included the average capacity factor of PV generation $\overline{\text{PV}}$ and wind power $\overline{W}$ on cost. Additionally, we included the variable l^{season} that indicates if CO_2 supply is stretched out during the whole year or if it is concentrated in a few months. We first derive the cumulative sum of the timeseries of the CO_2 stream minus the average CO_2 stream, i.e. $S_T^{\text{CO2}} = \sum_{t=1}^{T} \text{CO2}_t - \overline{\text{CO2}}$. We then determine at which point in time t this timeseries has its maximum and its minimium. The number of days between these two points in time measures the length of the period. To determine parameters of the regression model, we used a solar-wind scenario with average input cost assumptions ((see Supplementary Table 4 for details on the choice of techno-economic parameters). Using other scenarios in the regression changes the results quantitatively, i.e. coefficients are changed, but not qualitatively. The results are shown in Supplementary Table 5.

Supplementary note 4. Methods - Optimization model equations

The objective function for the cost minimization (eq. 1) sums up capacities times cost per unit of VRES generation, i.e. PV and Wind power, (x_vresBuild, resCost), the cost per unit of storage systems (x_storageSize, storageCost), the cost per unit of electrolyzers (x_electrolyzerSize, electrolyzerCost) and the cost per unit of methanol synthesis unit (x_methanolSynthesisSize, methanolSynthesisCost). The subindex tech denotes technology which can be either photovoltaic or wind power, subtech denotes the sub-technology classification that can be IEC-II or IEC-III for wind turbines (see Supplementary Note 6 for details) and c denotes the type of storage that can be either electricity, H_2 or CO_2. An overview of the sets, parameters and variables used is provided in Supplementary Tables 6, 7 and 8 respectively.

$$\begin{aligned} \text{x\_cost} = \sum_{\text{tech}} \sum_{\text{subtech}} & \text{x\_vresBuild}(\text{tech}, \text{subtech}) \times \text{vresCost}_{(\text{tech},\text{subtech})} \\ & + \sum_{c} \text{x\_storageSize}_c \times \text{storageCost}_c \\ & + \text{x\_electrolyzerSize} \times \text{electrolyzerCost} \\ & + \text{x\_methanolSynthesisSize} \times \text{methanolSynthesisCost} \end{aligned} \tag{1}$$

The VRES generation per time step ($\text{x\_vresGeneration}_t$) is balanced with the installed capacity (x_vresBuild(tech, subtech)) times the production profile ($\text{vresOutput}_{(t,\text{tech},\text{subtech})}$) as shown in eq. 2. VRES generation is also balanced with instantaneously used power ($\text{x\_vresPowerToUse}_t$), inflows to battery storage ($\text{x\_storageInput}_{(t,\text{"electricity"})}$) and curtailment ($\text{x\_vresCurtail}_t$) as presented in eq. 3.

$$\begin{aligned} \text{x\_vresGeneration}_t = \sum_{t} \sum_{tech} \sum_{\text{subtech}} & \text{x\_vresBuild}_{(\text{tech},\text{subtech})} \\ & \times \text{vresOutput}_{(t,\text{tech},\text{subtech})} \quad \forall t \end{aligned} \tag{2}$$

$$\begin{aligned} \text{x\_vresGeneration}_t = \text{x\_vresPowerToUse}_t + & \text{x\_storageInput}_{(t,\text{"electricity"})} \\ & + \text{x\_vresCurtail}_t \quad \forall t \end{aligned} \tag{3}$$

The speed of charging ($\text{x\_storageInput}_{(t,c)}$) or discharging ($\text{x\_storageOutput}_{(t,c)}$) storage systems is limited by the installed capacity (x_storageSize_c) times a charging speed limit

(storageCapacityLimitPercentage$_c$) (eq. 4 and 5).

$$\text{x\_storageInput}_{(t,c)} \leq \text{x\_storageSize}_c \times \text{storageCapacityLimitPercentage}_c \quad \forall t, c \tag{4}$$

$$\text{x\_storageOutput}_{(t,c)} \leq \text{x\_storageSize}_c \times \text{storageCapacityLimitPercentage}_c \quad \forall t, c \tag{5}$$

Equation 6 shows how the state of charge (x_soc$_{(t,c)}$) is balanced with the state of charge one time step before, accounting for temporal storage losses, with charging energy (x_storageInput$_{(t,c)}$), considering charging losses (storageEffcharg$_c$), and discharging energy (x_storageOutput$_{(t,c)}$).

$$\begin{aligned} \text{x\_soc}_{(t,c)} = (\text{x\_soc}_{(t-1,c)} \times (1 - \text{storageLoss}_c)) + (\text{storageEffcharg}_c \\ \times \text{x\_storageInput}_{(t,c)}) - \text{x\_storageOutput}_{(t,c)} \quad \forall t, c \end{aligned} \tag{6}$$

The sum of the electricity used to produce H_2 in this time step (electricityGenerationH2$_t$) is balanced with the sum of instantaneously used VRES electricity and the output of the electrical storage (eq. 7).

$$\text{electricityGenerationH2}_t = \text{x\_vresPowerToUse}_t + \text{x\_storageOutput}_{(t,electricity)} \quad \forall t \tag{7}$$

Moreover, the amount of CO_2 that is used for the production of methanol in any particular time step (eq. 8) is equal to the sum of the CO_2 stream from the ethanol production (co2Stream$_t$) and the output of the CO_2 storage (x_storageOutput$_{(t,\text{"CO}_2\text{"})}$) minus the CO_2 stored (x_storageInput$_{(t,\text{"CO}_2\text{"})}$).

$$\begin{aligned} \text{x\_co2ToMethanol}_t = \text{co2Stream}_t + \text{x\_storageOutput}_{(t,\text{"CO}_2\text{"})} \\ -\text{x\_storageInput}_{(t,\text{"CO}_2\text{"})} \quad \forall t \end{aligned} \tag{8}$$

The amount of H_2 (x_h2$_t$) is determined in two different equations (eq. 9 and eq. 10). It is equal to the electricity use of the electrolyzer (electricityGenerationH2t) multiplied by the efficiency of the electrolyzer (h2Eff). It's level is limited by the electrolyzer size (x_electrtolyzerSize) multiplied by its efficiency.

$$\text{x\_h2}_t = \text{electricityGenerationH2}_t \times h2Eff \quad \forall t \tag{9}$$

$$\text{x\_h2}_t \leq \text{x\_electrolyzerSize} \times \text{h2Eff} \quad \forall t \tag{10}$$

Similarly the amount of methanol in a particular time step (x_methanol$_t$) is limited on the one side (eq. 11) by the size of the methanol synthesis installation (x_methanolSynthesisSize) and on the other side (eq. 12) by the methanol synthesis efficiency (methanolSynthesisEff) multiplied by the amount of H_2 that can be transformed into methanol in that time step (x_h2ToMethanol$_t$). The latter is also equal to the sum of H_2 produced in that time step (x_h2$_t$) and the difference between charge (x_storageInput$_{(t,\text{"h}_2\text{"})}$) and discharge (x_storageOutput$_{(t,\text{"h}_2\text{"})}$) of the H_2 storage (eq. 13).

$$\text{x\_methanol}_t \leq \text{x\_methanolSynthesisSize} \quad \forall t \tag{11}$$

$$\text{x\_h2ToMethanol}_t \times \text{methanolSynthesisEff} = \text{x\_methanol}_t \quad \forall t \tag{12}$$

$$\text{x\_h2ToMethanol}_t = \text{x\_h2}_t + \text{x\_storageOutput}_{(t,\text{"h}_2\text{"})} - \text{x\_storageInput}_{(t,\text{"h}_2\text{"})} \quad \forall t \tag{13}$$

Finally, eq. 14 presents the restriction for the transformation of CO_2 into methanol (x_co2ToMethanol$_t$). It depends on the amount of H_2 that can be transformed into methanol at a particular time step and the proportion between CO_2 and H_2 for each Methanol unit (balanceCO2H2).

$$\text{x\_h2ToMethanol}_t \times \text{balanceCO2H2} = \text{x\_co2ToMethanol}_t \quad \forall t \tag{14}$$

Supplementary note 5. Methods - Sugarcane facility data set

The Brazilian sugarcane ethanol industry is highly dynamic and dependent on local regulation, national and international markets. While there are companies that have been in the market for decades, ethanol-producing installations are commissioned, re-commissioned and closed regularly. Furthermore, the production in each installation is not only conditioned by the seasonality of the sugar cane and weather, but also by the changes in prices of fuels and sugar at the national and international level. The consequence is that there is no single or consolidated data set on ethanol generation plants in Brazil. Previous studies modeling Brazilian ethanol production avoided the problem by either denying it or working only with data of one single exemplary installation. However, the spatial location and the time series of CO_2 emissions for each ethanol plant are necessary to properly account for the integration of variable renewables in methanol production.

The three official sources for data of the Brazilian ethanol industry are the Energy research company (EPE–Empresa de Pesquisa Energética), the National Agency of Petroleum, Natural Gas and Biofuels (ANP–Agência Nacional Do Petróleo, Gás Natural e Biocombustíveis) and the Ministry of Agriculture (MAPA–Ministério da Agricultura, Pecuária e Abastecimento). These provide lists of installations but differ not only in the number of reported installations but also in the provided attributes or the values of attributes. We, therefore, constructed a synthetic data set that consolidates the data of the available sources. The EPE data set (381 installations) was used as a basis since it is the only one providing geographic coordinates for the installations. We confirmed the existence and installed capacity of the installations using the ANP data set (360 installations including not only sugar cane but also corn, rice and soy), which was matched in a semi-automatic fashion supported by similarity ratings on the names of the installations as well as by proximity assessments. The ANP data set provides addresses, which were georeferenced for that purpose. The existence of the installations was corroborated again using the MAPA data set and in case of doubt about the match of the installation between the three previous data sets, a manual online search was conducted for those cases.

The synthetic data set includes 339 installations that run mainly on sugarcane. Based on ANP, the installations have average daily generation capacities of 365 m^3 and 676 m^3 for anhydrous ethanol and hydrated ethanol, respectively. There are however large differences between installations and the largest ones reach processing capacities of 1,710 m^3 day^{-1} of anhydrous ethanol and 2,800 m^3 day^{-1} of hydrated ethanol. Hydrated ethanol is composed of approximately 96% ethanol and 4% water while anhydrous ethanol contains at least 99.7% ethanol. The distillation process is common to both types of ethanol but the production of anhydrous ethanol requires an additional dehydration step to reduce the water content[26]. Considering this, we rated the processing capacity of each installation as the maximum value of the capacities for hydrous and anhydrous ethanol.

Apart from the location, the daily processing capacities, the state, the municipality and the type of biomass processed by each plant, there is not much public official information available on the installations. Based on EPE[27], we could confirm that only two of the installations have second-generation ethanol production and identify these installations in the data set. To approximate the sugar cane harvesting area of each installation, we relied on statistics of the Companhia Nacional de Abastecimento (CONAB) for each state. We calculated the average of harvested area for the last five years and distributed it by installation based on the share of ethanol processing capacity of each installation when compared to the sum of processing capacity of all installations in a particular state. The majority of sugarcane to ethanol production in Brazil is concentrated in the state of São Paulo, which has a number of installations and installed ethanol processing capacities larger than the next six states combined (Supplementary Fig. 1). From 339 installations in the consolidated data set, 145 are located in this state, with a total processing capacity of 107,348 m^3 of ethanol per day. This is followed by the state of Goiás, which hosts 36 installations, but is also home of several of the largest installations in the country with processing capacities of up to 2,800 m^3 ethanol per day. These states are followed in number of installations by the neighbouring states of Minas Gerais, Paraná and Mato Grosso do Sul, which have installed capacities for ethanol production of 34,210, 21,882, 13,460 and 22,385 m^3 day^{-1}, respectively.

Supplementary note 6. Methods - VRES installation capacity factors and footprints

Supplementary Fig. 11 and 12 present the average weekly capacity factors for solar PV (Photovoltaics) and wind power installation at all locations.

The tables associated to this note include the results of the assessment of Brazilian photovoltaic installation footprints (Supplementary Table 9), the corresponding descriptive statistics (Supplementary Table 10), and the footprints of installations reported in the scientific literature (Supplementary Table 11). In Brazil, we measured the extent of seven locations from the ANEEL power plants database[28]. The footprints are in a similar range to installations reported in the scientific literature. For the panels only, values of 7.1 to $14\,m^2$ kWp^{-1} were found in the literature, while for the impact of total systems, depending on the type of system, land-use values between 15.4 and $36.4\,m^2$ kWp^{-1}, except for one extreme case of $50\,m^2$ kWp^{-1}, were found.

References

[1] Vartiainen, E., Masson, G., Breyer, C., Moser, D. & Medina, E. R. Impact of weighted average cost of capital, capital expenditure, and other parameters on future utility-scale pv levelised cost of electricity. *Progress in Photovoltaics: Research and Applications* **28**, 439–453 (2020).

[2] Danish-Energy-Agency & Energinet. Technology data -energy plants for electricity and district heating generation. Tech. Rep., Danish Energy Agency (2016).

[3] Armijo, J. & Philibert, C. Flexible production of green hydrogen and ammonia from variable solar and wind energy: Case study of chile and argentina. *International Journal of Hydrogen Energy* **45**, 1541–1558 (2020).

[4] Hannula, I. Co-production of synthetic fuels and district heat from biomass residues, carbon dioxide and electricity: Performance and cost analysis. *Biomass and Bioenergy* **74**, 26–46 (2015).

[5] Papadias, D. & Ahluwalia, R. Bulk storage of hydrogen. *International Journal of Hydrogen Energy* **46**, 34527–34541 (2021).

[6] Decarre, S., Berthiaud, J., Butin, N. & Guillaume-Combecave, J.-L. CO2 maritime transportation. *International Journal of Greenhouse Gas Control* **4**, 857–864 (2010).

[7] Aspelund, A., Tveit, S. P. & Gundersen, T. A liquefied energy chain for transport and utilization of natural gas for power production with CO2 capture and storage – part 3: The combined carrier and onshore storage. *Applied Energy* **86**, 805–814 (2009).

[8] Øi, L. E. *et al.* Simulation and cost comparison of CO2 liquefaction. *Energy Procedia* **86**, 500–510 (2016).

[9] Svensson, R., Odenberger, M., Johnsson, F. & Strömberg, L. Transportation systems for CO2—application to carbon capture and storage. *Energy Conversion and Management* **45**, 2343–2353 (2004).

[10] Lauri, K., Jouko, R., Nicklas, N. & Sebastian, T. Scenarios and new technologies for a north-european CO2 transport infrastructure in 2050. *Energy Procedia* **63**, 2738–2756 (2014).

[11] Kaarstad, O., Hustad, C.-W., Nissen, K., Coleman, D. L. & Lekva, H. Delivering co2 to gullfaks and the tampen area - co2.no. Tech. Rep., Elsam A/S, Kinder Morgan CO2 Company L.P. and New Energy, Statoil (2003).

[12] Christensen, A. Assessment of hydrogen production costs from electrolysis: United states and europe. *Internation Council on Clean Transportation* 73 (2020).

[13] Mallapragada, D. S., Gençer, E., Insinger, P., Keith, D. W. & O'Sullivan, F. M. Can industrial-scale solar hydrogen supplied from commodity technologies be cost competitive by 2030? *Cell Reports Physical Science* **1**, 100174 (2020).

[14] Brändle, G., Schulte, S. & Schönfisch, M. Estimating long-term global supply costs for low-carbon hydrogen. *Institute of Energy Economics at the University of Cologne Working Paper* (2020).

[15] IRENA. *Hydrogen: A renewable energy perspective* (International Renewable Energy Agency, 2019).

[16] Powerfuels, G. A. Carbon sources for powerfuels production. Tech. Rep., Deutsche Energie-Agentur GmbH (2020).

[17] Pilorgé, H. *et al.* Cost analysis of carbon capture and sequestration of process emissions from the u.s. industrial sector. *Environmental Science & Technology* **54**, 7524–7532 (2020).

[18] CO_2-EOR. Capturing and utilizing co_2 from ethanol: Adding economic value and jobs to rural economies and communities while reducing emissions. Tech. Rep., State CO_2-EOR Deployment Work Group (2017).

[19] Hernandez, R. R., Hoffacker, M. K. & Field, C. B. Land-use efficiency of big solar. *Environmental Science & Technology* **48**, 1315–1323 (2014).

[20] Denholm, P. & Margolis, R. M. Land-use requirements and the per-capita solar footprint for photovoltaic generation in the united states. *Energy Policy* **36**, 3531–3543 (2008).

[21] Ong, S., Campbell, C., Denholm, P., Margolis, R. & Heath, G. Land-use requirements for solar power plants in the united states. Tech. Rep., NREL (2013). URL `http://www.osti.gov/servlets/purl/1086349/`.

[22] Wirth, H. Recent facts about photovoltaics in germany. Tech. Rep., Fraunhofer ISE (2020). URL `https://www.pv-fakten.de`.

[23] Atikol, U., Abbasoglu, S. & Nowzari, R. A feasibility integrated approach in the promotion of solar house design. *International Journal of Energy Research* **37**, 378–388 (2013).

[24] Prussi, M., De Prada, L., Edwards, R., Padella, M. & Yugo, M. *JEC well-to-tank report V5: JEC well to wheels analysis : well to wheels analysis of future automotive fuels and powertrains in the European context.* (Joint Research Centre (European Commission), LU, 2020).

[25] Methanex-Corporation. Methanol pricing (2020). URL `https://www.methanex.com/our-business/pricing`. [Online; accessed 2020-12-11].

[26] Holler Branco, J. E., Holler Branco, D., Aguiar, d. E. M., Caixeta Filho, J. V. & Rodrigues, L. Study of optimal locations for new sugarcane mills in brazil: Application of a minlp network equilibrium model. *Biomass and Bioenergy* **127**, 105249 (2019).

[27] EPE. Cenários de oferta de etanol e demanda de ciclo otto 2020-2030. Tech. Rep., Empresa de Pesquisa Energética (2019).

[28] ANEEL. Siga - sistema de informacões de generacão da aneel. Tech. Rep., Agência Nacional de Energia Elétrica (2020).
