# Supplementary material for: Pathway to a land-neutral expansion of Brazilian renewable fuel production
Source: Nat Commun. 2022 Jun 7;13:3157. doi: 10.1038/s41467-022-30850-2 (PMC9174478; doi:10.1038/s41467-022-30850-2)
Supplement: Supplementary file 3 — Reporting Summary [file 41467_2022_30850_MOESM3_ESM.pdf]

nature portfolio

Corresponding author(s): Ramirez Camargo, Luis and Schmidt, Johannes

Last updated by author(s): Apr 20, 2022

Reporting Summary

Nature Portfolio wishes to improve the reproducibility of the work that we publish. This form provides structure for consistency and transparency in reporting. For further information on Nature Portfolio policies, see our Editorial Policies and the Editorial Policy Checklist.

Statistics

For all statistical analyses, confirm that the following items are present in the figure legend, table legend, main text, or Methods section.

n/a	Confirmed	
☐	☒	The exact sample size (n) for each experimental group/condition, given as a discrete number and unit of measurement
☒	☐	A statement on whether measurements were taken from distinct samples or whether the same sample was measured repeatedly
☒	☐	The statistical test(s) used AND whether they are one- or two-sided<br>*Only common tests should be described solely by name; describe more complex techniques in the Methods section.*
☒	☐	A description of all covariates tested
☒	☐	A description of any assumptions or corrections, such as tests of normality and adjustment for multiple comparisons
☐	☒	A full description of the statistical parameters including central tendency (e.g. means) or other basic estimates (e.g. regression coefficient) AND variation (e.g. standard deviation) or associated estimates of uncertainty (e.g. confidence intervals)
☒	☐	For null hypothesis testing, the test statistic (e.g. F, t, r) with confidence intervals, effect sizes, degrees of freedom and P value noted<br>*Give P values as exact values whenever suitable.*
☒	☐	For Bayesian analysis, information on the choice of priors and Markov chain Monte Carlo settings
☒	☐	For hierarchical and complex designs, identification of the appropriate level for tests and full reporting of outcomes
☒	☐	Estimates of effect sizes (e.g. Cohen's d, Pearson's r), indicating how they were calculated

Our web collection on statistics for biologists contains articles on many of the points above.

Software and code

Policy information about availability of computer code

Data collection	Most data was obtained from third party repositories or publications. The land footprints of Wind and PV power installations were calculated using google maps
Data analysis	GAMS 29.1.0 is used for the optimization, Python 3.9 is used for the resources simulations and general data processing and R 4.0.3. is used for statistics and figures. Conda environments including all dependencies are provided in the Zenodo repository

For manuscripts utilizing custom algorithms or software that are central to the research but not yet described in published literature, software must be made available to editors and reviewers. We strongly encourage code deposition in a community repository (e.g. GitHub). See the Nature Portfolio guidelines for submitting code & software for further information.

Data

Policy information about availability of data

All manuscripts must include a data availability statement. This statement should provide the following information, where applicable:
- Accession codes, unique identifiers, or web links for publicly available datasets
- A description of any restrictions on data availability
- For clinical datasets or third party data, please ensure that the statement adheres to our policy

The full set of necessary input data for the optimization model and the final results presented in all figures are available under an open license on Zenodo (DOI: 10.5281/zenodo.6471331). Raw ERA5 and ER5-land data can be downloaded from https://cds.climate.copernicus.eu/#!/home. Similarly, the GWA data can be obtained from https://globalwindatlas.info/download/gis-files. Corresponding download scripts are also provided in the repository. Moreover, we obtained raw data from ANEEL, EPE, CONAB, ANP and MAPA from https://www.gov.br/aneel/pt-br, https://www.epe.gov.br/pt, https://www.conab.gov.br/, https://www.gov.br/anp/pt-br, and https://www.gov.br/pt-br/orgaos/ministerio-da-agricultura-pecuaria-e-abastecimento to compile our data set about existing ethanol sugar-cane

facilities. The data sets are partially geo-restricted (i.e. they require a Brazilian IP address) and we do not have the rights to re-distribute them. However, we provide a CSV file which contains the consolidated information necessary to run our code in the repository.

Field-specific reporting

Please select the one below that is the best fit for your research. If you are not sure, read the appropriate sections before making your selection.

☐ Life sciences ☐ Behavioural & social sciences ☒ Ecological, evolutionary & environmental sciences

For a reference copy of the document with all sections, see nature.com/documents/nr-reporting-summary-flat.pdf

Ecological, evolutionary & environmental sciences study design

All studies must disclose on these points even when the disclosure is negative.

Study description	We use an optimization model (techno-economic-environmental) to assess the feasibility of a pathway to decrease the land use impact of fuel production at ethanol plants
Research sample	We do not sample, but calculate our pathway for all sugarcane ethanol producing plants in Brazil. For this we generate a consolidated data set of all sugar-cane ethanol producing plants in Brazil based on data obtained from the Empresa de Pesquisa Energetica, Agencia Nacional do Petroleo, Gas, Natural e Biocombustiveis and the Ministerio da Agricultura, Pecuaria e Abastecimento
Sampling strategy	As we do not sample we do not use any sample strategy
Data collection	Data was obtained from third party sources. Most data downloads are automatic and can be reproduced using our code. The compilation of the ethanol facilities includes manual downloads, described in detail in the supplementary material of the manuscript.
Timing and spatial scale	Data on Brazilian sugarcane ethanol facilities was collected between the second half of 2019 and the first half of 2020. All other data sets were updated upon final submission of the accepted manuscript.
Data exclusions	Data on sugarane ethanol installations which was not confirmed by more than one of the official sources was excluded. No other exclusions ocurred.
Reproducibility	References to all data sources are provided and a repository with the code for data treatment is available under:
Randomization	Does not apply. We worked with the full data set of installations.
Blinding	Does not apply. We worked with the full data set of installations.

Did the study involve field work? ☐ Yes ☒ No

Reporting for specific materials, systems and methods

We require information from authors about some types of materials, experimental systems and methods used in many studies. Here, indicate whether each material, system or method listed is relevant to your study. If you are not sure if a list item applies to your research, read the appropriate section before selecting a response.

Materials & experimental systems

n/a	Involved in the study	
☒	☐	Antibodies
☒	☐	Eukaryotic cell lines
☒	☐	Palaeontology and archaeology
☒	☐	Animals and other organisms
☒	☐	Human research participants
☒	☐	Clinical data
☒	☐	Dual use research of concern

Methods

n/a	Involved in the study	
☒	☐	ChIP-seq
☒	☐	Flow cytometry
☒	☐	MRI-based neuroimaging
